# Foods tabooed for pregnant women in Abala district of Afar region, Ethiopia: an inductive qualitative study

**DOI:** 10.1186/s40795-017-0159-x

**Published:** 2017-05-02

**Authors:** Znabu Hadush, Zewdie Birhanu, Mulugeta Chaka, Haylay Gebreyesus

**Affiliations:** 1 Department of Public Health, Samara University, Samara, Ethiopia; 2 Department of Health Education and Behavioral Sciences, Jimma University, Jimma, Ethiopia; 3 Department of Public Health, Axum University, Axum, Ethiopia

**Keywords:** Food taboos, Pregnant women, Afar, Qualitative study, Ethiopia

## Abstract

**Background:**

Food taboo is contributing substantially to malnutrition for pregnant women by restricting and limiting the frequency and variety of foods most of which are nutritious and easily accessible. The practice is common in developing countries and most of the food taboos in East Africa fall on the women and most unfortunately on the pregnant. Foods of animal products, which are the main sources dietary energy of pastoralist communities, are often prone to the practice of food taboos. Nonetheless, the existence of the practice in Ethiopian pastoralist communities, the communities whose way of life is mostly nomadic and based on tending of herds or flocks, is not investigated yet. Therefore, the current study aimed to explore foods tabooed for pregnant women and the reasons behind the practice if exists in Abala district of Afar region, Ethiopia.

**Methods:**

Exploratory qualitative study was conducted inductively involving homogeneous participants in four focus group discussions and eight key informants in individual in-depth interview who were purposively selected in Aballa district from March 1 to 30, 2016. A semi-structured interview guide was used to collect the data. The investigators audiotaped focus group discussions and interviews and then transcribed them verbatim. Finally, the transcribed data were imported to Atlas.ti 7 software for coding. Analysis was done inductively. Triangulation and peer debriefing were applied to assure data quality.

**Results:**

The study revealed that foods tabooed for pregnant women were 1) Eating a large amount of food of any type, 2) fatty foods like meat, milk and yoghurt, 3) Foods that are not in liquid form such as different types of bread and 4) cool/cold foods such as cold milk, cold meat and cold water. The reasons mentioned to adhere with the foods taboo for pregnant women were to avoid difficulty to deliver the fetus, to prevent disease like Gastritis, Diarrhea, Typhoid and skin discoloration of the fetus. Besides, inconveniences like abdominal cramp were reported as reasons to adhere the foods tabooed.

**Conclusions:**

Pregnant women in Aballa district avoid eating numerous accessible foods because the foods are believed as tabooed for them. Further studies that focus on the extent of food taboo and uncovering the understanding on how it is being practiced were recommended.

## Background

Food taboo is any consideration of food items by the society as improper or unacceptable that arises mainly based on religious, cultural, historical and social principles. According to the earlier literature, the consequence of not adhering to an established food taboo is always defined by the society as it causes illness or death, which is similar across different communities of the world [1–3]. It either may govern the completely human life cycle or may be associated with special events such as pregnancy, childbirth, and lactation. Evidence shows that food taboo accounts largely to maternal and fetal malnutrition during pregnancy [4, 5].

The practice of food taboo is high in developing countries though there is inconsistency on which food is considered as tabooed and the attached reason from society to society. About 49% of pregnant mothers in India [6], 69% in Tanzania [7], 49% in Ghana [8], 27.5% in Hadya zone, Ethiopia [9], 49% in Shashemene district of Ethiopia [10] reports avoidance of at least one food item during pregnancy. An ethnographic study from Madagascar shows that 77% of stories about the origins of food taboos are related to health and well-being [5].

However, restriction and or inadequate intake of some food items during the period of pregnancy for different reasons limit the frequency and variety of food, which makes consequences of malnutrition most awful [11–14]. For the reason that women’s energy requirements remain high during pregnancy and given the detrimental impacts of inadequate maternal nutrition on both gestational and neonatal outcomes, the health of pregnant can be affected by their adherence to food taboos [1, 15, 16]. Besides, evidence also shows that malnutrition in women reduces their ability to work, which in turn creates ripple effects for the women, their family and the country. Furthermore, it is the underlying cause of 3.5 million preventable child and maternal deaths a year and 35% of the disease burden in children younger than five years worldwide [17–19].

Literature also shows that most of the foods either restricted or tabooed are inexpensive, nutritious and good for both the mother and the fetus while the reasons stated for each tabooed food have no mostly scientific explanation [7]. Despite of their share up to more than 50% in pastoralist communities’ dietary energy requirements (DER) in sub-Saharan Africa, foods of animal origin are prone to cultural food beliefs and taboos [20].

Given the possible social sanctions for disobedience to food taboos, literature indicates that the odds of adherence to food taboo during pregnancy tend to be higher among women, which are teenager while they give birth, prim-gravidi, attained low educational status, and from low-income families [21, 22]. In these perspectives, most of the women in Afar region are illiterate, with low socio-economic status, low decision-making power on household income, and face a risk of harmful traditional practices like female genital mutilation, early marriage and abduction. Moreover, most of them suffer from acute malnutrition that made them the ever-thinnest women population in Ethiopia [23–25].

Despite the socio-demographic characteristics of women in pastoralist communities of Afar could make them prone to the risk of food taboo, the existence of the practice is not well studied. Therefore, this study aimed to close this gap by exploring the practice of food taboos among pregnant in Abala district of Afar region. The research questions for the study were 1) what are the foods tabooed for pregnant women in Abala district of Afar Region, and 2) what are the reasons behind the adherence to food taboo among pregnant women if exist.

## Methods

### Study setting

#### Location

Aballa district is one of the districts in Kilbet Rasu zone of Afar region in which Aballa town is the administrative center of the zone. The remain districts under the zone are Afdera, Berhale, Dallol, Erebti, Koneba and Megale. Abala is located 775 Km North from Addis Ababa, the capital of Ethiopia, and 489 Km North West of Samara town, the Capital of Afar. Based on figures from the Central Statistical Agency in 2005, Abala has an estimated total population of 6601 of whom 3448 are men and 3153 are women by the year of 2016. The district constitute of 14 kebeles (Smallest administrative unit).

#### Socio-demographics

Majority of the Afar population is Muslim in religion and rural in residence. Regarding educational status, 74% of females have never attended school in Afar region. The region is among the regions in Ethiopia with high total fertility rate (5.7) and the proportion of mothers getting pregnant each year among women of reproductive age groups is twofold higher than that of Ethiopia [23].

#### Economic characteristics and malnutrition

Based on 2007 Ethiopian central statistical agency, about 30% of its people are pastoralists (CSA, 2007). Afar region in which the study setting is found in, is characterized by being the leading region to have high proportion of population with lower wealth quintile (60%) According to the Mini Ethiopian Demographic Health Survey report (MEDHS) of the year 2014, about 44% of the women in Afar region, in which the study setting is found in, suffer from acute malnutrition; which made them the ever-thinnest women of any region in the country, while 35% experience high anemia. Meanwhile, 31% of under-five children in the region are severely stunted (chronic malnutrition) and 24% are wasted (acute malnutrition) which is two times and three times respectively higher than the national prevalence. Moreover, nearly half of under-five children are underweight in the region [10].

#### Culture

Regarding harmful traditional practices, women from Afar region are likely to pass through the experiences of FGM, child marriage and abduction and wife beating [26]. Afar society has customary laws, which bind all their people and have a clear, but unfortunately unfair, distribution of role among the family members. Men are the head of the family and represent the family at all level. However, grinding, food preparation, serving food, looking after goat and sheep, bearing, and rearing children are the responsibility of the women [27].

#### Health facility

Though there is two folds higher proportion of pregnant mothers among women of reproductive age groups in the region than the national proportion, utilization of maternal health and related services is still low. Only 8.5% of women of childbearing age use modern contraception, 29% of pregnant women receive antenatal care (ANC) from skilled provider and only 6% receive skilled delivery [23].

#### Study design

An exploratory community based qualitative study was conducted inductively from March 01 to may 10, 2016.

#### Population

All currently pregnant and lactating women and key informants residing in four purposively selected kebeles (smallest administrative unit) of Aballa district were source population of the studies. The kebeles were Ukri Gibi, Murga, Kaala and Adi Haremely. All currently pregnant women (gestational period of 3 months and above ascertained by self-report) and lactating women who are currently breastfeeding children of 2 years and below and elderly women and men were eligible for the study. Respected elderly members of the community for known social status (e.g. religious leader, clan/community/ethnic leader, traditional birth attendant) with potential information on the issue were eligible.

### Sample size and sampling procedure

Four Focus Group Discussions (FGDs) that each consisted of 6 to 8 homogeneous participants were conducted. The FGDs were conducted among male elderly, female elderly, pregnant and lactating women FGDs. Besides, eight individuals were purposively selected as key informants for the in-depth interview that included pregnant and lactating women, female elderly, male elderly, health extension workers, religious and community leaders. Purposive sampling technique was used to select study participants for FGDs and in-depth interviews (IDI). With the help of community health workers and leaders, study participants were selected for FGDs and IDIs. The criteria for recruiting were the potential relevance of the participants in delivering a wealth of information about food taboos for pregnant and lactating women. Indented to this, maximum variation technique was applied to include participants with variations of characteristics like educational status, residence and age. At each kebele, one FGD and two IDIs were conducted.

### Data collection methods and tools

The study adapted an English version of a semi-structured guide from a doctoral dissertation by Samson Korvah Arzoaquoi on “Common food taboos and beliefs during pregnancy in Yilo Krobo district, University of Ghana, Ghana, 2014 (Unpublished Article). The tool consisted of the following sub-sections: 1) list of food tabooed during pregnancy and lactating period, 2) the adherence level of pregnant and lactating women to the food taboos if exist, and 3) the reasons to adhere to the foods taboo. Then, the tool was contextualized, translated in the local language (Afar) and finally two sample interview were conducted in two other kebeles that were not included in the main study to pretest the tool for wordiness and cultural sensitiveness.

Focus group discussions followed by IDIs were employed to collect the data. For the FGDs, convenient location for most of the participants, suitable to sit for them in a circular way seeing one another a face-to-face and recoding is possible with minimum external disturbance was selected. The principal investigator (PI), a recorders/note taker and a translator facilitated each FGD and their roles were clearly defined before the FGD was conducted. The FGDs explored the general group norm on foods tabooed and the variation of views in the types of foods tabooed, in the level of adherences to and the reasons behind foods tabooed in the community. Preliminary analysis of each FGD was done before the next FGD. Hence, newly emerging insights and questions were added for clarification and depth in the followed FGD. The principal investigator conducted the eight IDIs for further depth of information and triangulation. The principal investigators (PI) conducted face-to-face interview with the help of the translator using the IDI guide. Data were recorded using tape recorder and the investigators took note including memos of participant’s behavior and contextual aspects to assure triangulation of the data with the record. The FGDs took a minimum of an hour and a half and the IDIs took a minimum of 45 min.

### Data analysis

The investigators analyzed the data side by side of data collection. For all independently recorded FGDs and IDIs, they transcribed verbatim after a minimum of three times repeated listening. The transcribed documents were imported in to Atlas.ti version for qualitative data analysis software version 7 for coding and analysis. The investigators then coded the respondent’s words, phrases, sentences and memos that were relevant to the area of the study. They systematically coded raw data openly and categorized the sub- themes under to their respective themes. Then, they created non-repetitive central themes that were constructed based on the natural meaning of categories. Finally, the investigators also cross-cheeked the themes emerged after analysis with the raw data and respective quotes in each category of the themes. Direct quotes of the participants were included in the write up of the findings. Moreover, in the overall process of data analysis, an inductive approach was applied.

### Data quality management and trustworthiness

Developed data collection tools were pretested in a similar context to maximize the validity of the tool. Probing and multiple data sources (FGD and IDI) were employed to collect the data. Throughout the study, bracketing the preconceptions of the investigators was employed to minimize the investigator’s bias and the risk of reactivity by the participants. The emerging findings of analysis were shared to experienced qualitative researchers for peer debriefing before synthesizing the final outputs.

#### Ethical consideration

Ethical review committee of Jimma University approved the research topic and permission to conduct the study was obtained from Afar Regional State Health Bureau and Abala district Health Offices. Participants were assured of confidentiality and written informed consent was sought after explaining the aim of the study.

## Result

Twenty-nine study participants were involved in four focus group discussions (FGD). All participants of the FGDs and Key informants in-depth interview (IDI) were Muslim in religion. The maximum educational status attained lay under the range of no formal education up to grade six. Three of the FGDs were conducted in rural settings while the remained one was in urban setting (Table 1).

**Table 1:** Socio demographic characteristics of FGDs participants on foods tabooed for pregnant and lactating women in Aballa district, Afar region, Ethiopia, 2016

FGDs	Number of participants	Mean age of participants (Min, Max)	Number of children (Min, Max)	Educational level (Min, Max)	Kebele
Pregnant and lactating women FGD1	8	31(21,39)	2,7	0–3	Ukri Gibi
Pregnant and lactating women FGD2	8	24(19,41)	3,8	0	Murga
Elderly men FGD	7	45(35,65)	5,11	0–6	Kala
Elderly women FGD	6	47(38,55)	4,9	0	Adi Heremly

Regarding the in-depth interview, the saturation of information was reached after eight key informants were interviewed. All of them were Muslim in religion and their age ranged from 21 to 66 years. Besides, educational status of the key informants ranged from illiterate to grade ten (Table 2).

**Table 2:** Socio demographic characteristics of Key informants for foods tabooed for pregnant women in Aballa district of Afar region, Ethiopia, 2016

Participants	Sex	Age	Educational status	Role of the participant	Kebele
Participant 1	Female	23	10	Health extension worker	Ukri Gibi
Participant 2	Female	21	10	Health extension worker	Murga
Participant 3	Female	30	No read write	Pregnant women	Kala
Participant 4	Female	27	6	Lactating women	Adi Haremely
Participant 5	Female	35	No read write	Elder	Kala
Participant 6	Male	54	No read write	Elder	Murga
Participant 7	Male	66	4	Elder	Adi Haremely
Participant 8	Female	40	No read write	Traditional women	Ukri Gibi

### Foods tabooed for pregnant women

The study participants in Aballa district of Afar region reported foods that are solid in their structure, fatty in their content and cool foods as tabooed for pregnant. They revealed that women other than the pregnant could eat the foods. Furthermore, eating large amount of food was highly recognized as tabooed for pregnant women in the district.

#### Solid foods

According to the study participants, foods named as “Burkutta”, “Ambassha”, “Bahamo” and “Mengelle” are tabooed for pregnant women in Aballa district. All are forms of bread prepared locally using ether firewood or solar as source of heat. Almost all respondents reported that solid foods are tabooed for pregnant. Thirty-four year old pregnant FGD participant stated,

*“Burkutta and Mengelle are not good foods for pregnant. She should abstain eating these foods until she gives birth. Personally, I do not like to eat these foods myself always during my pregnancy. I also advise others to avoid eating the foods.”*

Another 29 years pregnant also explained, *“Pregnant women should eat soft foods while the solids could be eaten during lactating after a month and a half (They call this period as “Elalo”) after giving birth”*. While another 22-year lactating woman FGD participant added as *“pregnant women would not eat solid foods like ‘Burkutta’ and ‘Bahamo’ and including solid ‘injera’ during her time of pregnancy.”*

A 56-year-old woman elderly also mentioned that pregnant women should not eat roasted seed (“Kalo”) saying*“… it would be painted at the head of the baby if eaten because it would not be dissolved till she give birth”.* A 35-year community elder key informant also mentioned what pregnant women should eat as; *“They would not eat solid foods. They can drink cow milk eat porridge with butter during their pregnancy.”*

Additionally, a 30 year pregnant key informant who had ever give birth four times before the current pregnancy also stated that *“Foods that are prohibited for pregnant women are ‘Burkutta’, which is solid bread, ‘Mengelle’ and ‘Behamo’ (bread forms made of wheat on stone with solar or fire).*

#### Fatty foods

The participants also reported that a pregnant woman should avoid eating foods that are associated with the high-fat content. They specified meat, camel milk and yoghurt/ “Ergo” as highly fatty foods. They call the foods as “good foods”, and pregnant woman should avoid eating these “good foods” to prevent the fetus from being large. A 27-year lactating key informant stated, “*Pregnant women should avoid particularly eating camel meat while the meat of other sources such as cow, goat and sheep are good for the pregnant. Particularly meats of newly born goats and sheep are good for the mother. Cow milk is also good for pregnant women.”* While 66 year elder key informant reflected a different view on meat, *“pregnant women should avoid eating meat and yoghurt at all till she would give birth.”* Another 40-year traditional birth attendant (TBA) also agreed on the view of the elder and she explained it as, *“Pregnant women should be strongly advised to abstain from eating meat during their pregnancy. When she reaches at her seventh and eighth month of her pregnancy she should completely stop eating milk, meat and some other good foods.”*

#### Cool foods

According to the current study, pregnant women in Aballa district avoid eating foods that are not warm and heated. The study participants reported that pregnant women should avoid eating cold foods during the period of their pregnancy that includes mainly cool meat, cool milk, yoghurt cheese and cool water. A 41 years old male elderly FGD participant stated this situation as, *“Pregnant women should avoid foods that are not heated with fire before eating. Milk and yoghurt should be heated so that the mother would not develop the disease if she drinks it.”*

Another 45 –year old FGD men elderly explained, *“Pregnant women should not make stay cooked meat for longer hours because they would be cool. They should consume them immediately as they are hot and warm. If not they should heat it again before eating.”* In line with these views, A 66 –year old man elderly key informant also mentioned, *“Foods that do not pass through fire are not good for pregnant women to eat. Mostly cool milk, yoghurt and cheese are prohibited…”.*

Kebele Health Extension Worker (HEW) aged 21 and worked for six years in the district also explained this phenomenon as: *“Most pregnant women prefer to consume warm foods such as porridge, tea, coffee and hot milk and avoid cool foods like yoghurt and cheese during their period of pregnancy. This is also the case for the lactating women.”*

Another FGD participant, 32 years aged pregnant women also added that *“… Cool foods including cool water should be avoided from eating through the whole period of pregnancy and the first six weeks of lactation to avoid diseases associated to her abdomen…”* While another 30-year old currently pregnant during the time of data collection also mentioned,

*“Foods that are not heated are not good for pregnant and lactating women. Though I have never seen lactating women having abdominal disease/“Medalyta” because she drinks cool foods, I know pregnant women having that disease because she eats cool milk. I myself also have experienced it two months ago.”*

Similar to the most of the respondents, a 49-year old FGD participant reflected a view in the issue, *“Camel Milk, cow milk and goat milk is good for pregnant women if it is new. However, if it have stayed for a days it will cause gastritis and heartburn.”*

#### Much food

The study participants repeatedly mentioned that a pregnant woman should abstain from eating much during pregnancy. According to the participants, a pregnant should try to limit her diet in quantity and frequency to prevent the fetus from becoming very large thus; she would not have difficulty and bleeding during delivery. Almost all FGD and IDI participants except a 27-year old lactating female mentioned that eating much food of any type including water as tabooed for pregnant. FGD participants agreed on when to be said, “A pregnant eats much food” as “If she eats an amount of food as equal as she always eats when she is not pregnant.”

Forty-year-old traditional birth attendant explained this situation as:

*“… A pregnant woman should avoid eating much all the way through the period of her pregnancy so that the fetus will not become large. Nevertheless, if she eats too much as usual, firstly the fetus will become too large to for delivery. Secondly, she will experience severe bleeding during delivery.”*

Another 43 old pregnant FGD participant reported, *“A pregnant women should reduce the number of meals she eats daily as she reaches the late months of her pregnancy*.” While other pregnant women added to this view *“If the mother is too fatty, she should reduce her fat to keep the fetus from being large. She could fetch water; collect firewood; and looking after goats by walking a long distance. This could help her to become thin.”*

Furthermore, pregnant women in Aballa district tend to increase the adherence of the food taboo as they become close to the end of their pregnancy. FGD participant from Murga kebele explained it as, *“If a pregnant woman is in the last months of her pregnancy, she should decrease the amount of all types of food items she eats in general, while meat, in particular, should be completely avoided.”*

### Reasons to adhere to food taboo for pregnant women

According to the study participants, the reasons for pregnant women to adhere to food taboo in Aballa district were difficulty of delivery because the fetus would becomes large in size, fear of disease for the mother and skin discoloration of the baby after birth.

#### Difficulty in delivering the fetus

According to the participants, the problem of difficulty to give birth is mainly due to the large size of the fetus attributed to eating much food of all type. Additionally, the frequent presence of fatty foods in their diet during pregnancy was reported as a contributor. Hence, eating down is recommended by the community to make the size of the fetus keep small as much as possible so that it will help to ease the difficulty she would experience during giving birth. Another 42 years old elderly men FGD participant also stated his agreement to pregnant women’s avoidance of “good foods” saying it as, *“I believe they should avoid meat, milk, yoghurt. The pregnant mothers should also abstain from eating a much amount of food including water to prevent the fetus from getting large to reduce the risk of a long labor, bleeding during labor. This problem is usual in our community; most women who ever give birth have experience of this problem.”*

Regarding the occurrence of the problem, A community leader also stated the level of the difficulty she might face as, *“In our community, it is common that a pregnant woman experiences a long period of labour and severe pain during delivery. She may take three to four days of labour. To my observation, I have seen pregnant women experiencing a problem during delivery including severe bleeding.”*

#### Sever bleeding during labour and delivery

The study participants also mentioned that In addition to eating much food and fatty food types, severe bleeding during labour and delivery could occur due to eating solid foods. The community believes that solid foods, particularly different forms of bread, cause severe bleeding during labour and delivery. Sixty-six men elderly key informant explained it, *“The pregnant would not have the power to dissolve solid foods such as “Burkutta”. Therefore, the foods could stay at her abdomen till she gives birth thus makes her to bleeding during delivery.”*

#### Prevent disease

Next to difficulty during delivery, preventing disease for the pregnant was the most repeatedly mentioned reason for obedience to food taboo for pregnant women. Gastritis was the most frequently mentioned disease followed by diseases or illnesses specified as diarrhea, vomiting, abdominal cramp, typhoid and heartburn. Participants further specified that solid foods are believed to cause gastritis (they call it as “Ali diduh”) to the pregnant because they are very sharp and hard to dissolve. Twenty-seven year old lactating female key informant also reported *“Burkutta and “Buhammo” cause gastritis (Ali diduh) to the pregnant. The foods make the pregnant to drink a lot amount of water thus causes gastritis and other diseases”*. She also added, *“Avoiding eating them helps them to avoid diarrhea, abdominal disease and vomiting for their child.”*

The reasons mentioned by the participants to avoid cool/cold foods (cool milk, cool meat, yoghurt and cheese) were that these causes bloody diarrhea, vomiting, abdominal disease and typhoid. A rural 54 years old key informant TBA also reported that she rarely sees a pregnant woman eating cool foods in her community that experienced diarrhea and abdominal cramp. She added her experience of diarrhea and vomiting as the result of drinking cool milk when she was a pregnant. Another 54 aged elderly male key informant stated his opinion on why pregnant women should avoid eating cold foods as, *“…regarding the cold foods, pregnant woman should avoid eating foods that are not hot. During her pregnancy period, she should not eat cool milk forms such as cheese because they cause diarrhea, abdominal disease and vomiting. They also can cause typhoid for the mother if frequently eaten.”*

The study participants also added diarrhea as among ill-health consequences of eating fatty foods such as meat and milk during pregnancy.

#### Skin discoloration

Few participants also reported that preventing discoloration of the skin of the baby, as the other reason to avoid eating fatty foods especially camel’s milk and camel’s meat during pregnancy. Though the FGD participants did not reach an agreement, a 25-year lactating woman mentioned it as, *“… If a pregnant woman eats fatty meat, the fat will be painted on the head of the fetus thus the head skin of the baby becomes yellow in color when born. Eating camel meat particularly makes the head skin yellowish”* Another 27-year old key informant, also reported *“The place of the head where that fat painted on would be mostly yellowish hair and sometimes bald. Camel milk and fatty meat are the main causes for balding”.*

Only a few participants mentioned the need to avoid eating roasted grains (they named it “Kollo”) during pregnancy explaining it could be painted on the head of the baby when born. A 30-year-old pregnant woman key informant stated it as, *“Roasted grains should not be eaten frequently by pregnant women. It does not cause a problem for the woman but it could be painted on the head of the baby. Sometimes it could cause an ulcer on the place that is painted often on the head.”* She also added the head of the baby as the part of the baby that could be painted and it rarely to happen on other body parts of the baby.

## Discussion

The current study tried to explore the foods tabooed for pregnant women and the reasons behind the practice of food taboo in Aballa district of Afar pastoralist community. According to the current study, “good foods” were reported as tabooed for pregnant women, as most of the foods were associated with the size of the fetus and the difficulty in delivering it. Hence, based on the community’s orientation, a fetus should be small to the minimum possible to reduce the risk of difficulty to deliver it. For this to happen, it was reported that pregnant women should avoid eating a large amount of food and fatty foods. Compliance to these foods tabooed was reported helpful to ease the problems attached to labour and delivery including severe bleeding.

This result is in line with other findings from Hadya zone and Shashemene district of Ethiopia and South Eastern Nigeria that reported women’s claim to difficult delivery as the result of increased size of the fetus due to consumption of nutritious foods [9, 10, 28, 29]. This may be due to lack of knowledge on the need for weight gain during pregnancy for the health of the fetus and the mother. Though it may not be the original reason to classify some food items as tabooed for pregnant women, the authors posit that the wide prevalent practice of female genital mutilation in Afar community may also contributes to sustain the thought, as the elastic nature of women’s vagina may be lost and thus increase the risk of difficulty to deliver the fetus. Evidence from Afar region previously revealed that the prevalence of female genital mutilation in the region ranges to 90% by 2012 [26].

In Aballa district, foods of animal sources including meat, milk and milk products were reported as tabooed for pregnant women for the main reason that they could make the fetus large because of their content. This result is similar in a finding found from Hadya zone of Southern Ethiopia that revealed milk and cheese as the most common tabooed foods for pregnant mother avoided by nearly half of the women in the zone [9]. A study from Shahsemene also reported milk and fatty meat as tabooed for pregnant women [10]. A study conducted in west Malaysia, Mid-west Nigeria and India supports this finding [5]. Though milk has a potential to contribute up to half of dietary energy requirements among pastoral communities [20], it is a strong food taboo for pregnant mothers in Aballa district of Afar region.

In addition to the myth of eating down and abstain from eating “good foods” for the pregnant women, the current study also shows that solid foods (like different bread forms) and cool foods (including milk, yoghurt, cheese, water and meat) were tabooed for pregnant and lactating women. The reasons reported were the solids are believed to cause gastritis and the colds are believed to predispose diseases like diarrhea, typhoid and abdominal cramp.

This finding seems in line with previously reported evidence that the pregnant women often face a large number of food proscriptions [30, 31]. Meanwhile, this evidence may underpin that food taboo is adding an additional challenge to maternal and child nutrition in the district. In synergy with other socio-demographic and economic factors, food taboo could pose a considerable risk for maternal malnutrition. Previous studies revealed that 60% of the population in Afar region is under lower wealth quintile, about 74% of women are illiterate and women have low decision power in household income. Besides almost every woman (90%) had experienced female genital mutilation once in their life [23, 24, 26, 27, 32, 33]. Therefore, it is anticipated that food taboo would make the women continue as disproportionally affected by malnutrition.

Regarding the reasons to avoid some of the food items for pregnant women, i.e. eating much, fatty foods, solid foods and cold foods) the reasons lay under three categories. The first is minimizing the risk of difficulty in delivering by shortening the duration of labour and reducing risk of excessive bleeding. The second is preventing diseases such as gastritis, diarrhea, typhoid and abdominal cramp. Other findings in the globe also repeatedly reviled illness and death as the consequence of not adhering to an established taboo [1–3, 5, 34].

The third reason was preventing skin discoloration of the infant when born. Similarly, a previous study in Shashemene district of Ethiopia reports that eating some vegetables during pregnancy would cause skin discoloration assuming that it would be plastered on the fetal head while born [10]. Despite the community reported such beliefs, neither documented evidence indicates that food eaten by the pregnant could be plastered at the skin of a fetus nor deviation from normal skin color like yellowish could be attributed to food eaten by the women while she is pregnant.

Among the reasons reported for adherence to the established food taboos, religious related orientations were not reported yet, which makes this finding different from a finding from Ghana district in which religious leaders either advice or remind pregnant women to keep compliant to the foods tabooed [35].

However, the study was not without limitation. As the study was exploratory and data were collected at a point of time/cross-sectional, the possibility of addressing sufficient depth and scope of explaining why the food taboos existed in the community may be limited.

## Conclusions

Foods tabooed for pregnant women were found to exist in Aballa district. Pregnant women avoid eating much food of any type and fatty foods to prevent the fetus get large and difficult to deliver. Foods that are in solid forms like bread were prohibited for pregnant assuming that it prevents diseases for the pregnant and the fetus. Likewise, they also avoid cool/cold foods, including cool milk, cool spice and cool water, to prevent diseases associated with the foods. Hence, food taboo could pose considerable risks of maternal malnutrition and their offspring. The current study uncover that most of reasons mentioned that why pregnant women need to adhere to the established food taboos are less likely to be grounded in the reality and mistaken. Besides, most of the explanations contradict with the need to increase frequency and diversity of foods during pregnancy to satisfy the increased energy requirement during pregnancy. Thus, strategic health communication that focuses correcting the wrong beliefs like pregnant women should eat less; some foods cause diseases pregnant and the fetus; and skin discoloration for fetus. Furthermore, longitudinal designs like ethnographic study could enable the scientific community and policy makers to understand the holistic perspective of food taboos in pastoral community in more depth. Besides, a representative quantitative study designs were also recommended assuming that it would determine the extent of the practice regarding food taboos.
